# Uranium-free X solution: a new generation contrast agent for biological samples ultrastructure

**DOI:** 10.1038/s41598-020-68405-4

**Published:** 2020-07-14

**Authors:** Aldo Moscardini, Sebastiano Di Pietro, Giovanni Signore, Paola Parlanti, Melissa Santi, Mauro Gemmi, Valentina Cappello

**Affiliations:** 1 NEST, Scuola Normale Superiore, Piazza San Silvestro 12, 56127 Pisa, Italy; 2 Dipartimento di Farmacia Università degli Studi di Pisa, Via Bonanno Pisano 6, 56126 Pisa, Italy; 3 Fondazione Pisana per la Scienza, Via F.Giovannini 13, 56017 San Giuliano Terme, PI Italy; 4 Child Health Institute of New Jersey and Department of Neuroscience and Cell Biology, Rutgers Robert Wood Johnson Medical School, 89 French Street, New Brunswick, NJ 08901 USA; 5 Istituto Italiano di Tecnologia, Center for Nanotechnology Innovation @NEST, Piazza San Silvestro 12, 56127 Pisa, Italy

**Keywords:** Biological techniques, Imaging, Electron microscopy, Bioinorganic chemistry, Organometallic chemistry, Imaging studies, Solution-state NMR

## Abstract

Biological samples are mainly composed of elements with a low atomic number which show a relatively low electron scattering power. For Transmission Electron Microscopy analysis, biological samples are generally embedded in resins, which allow thin sectioning of the specimen. Embedding resins are also composed by light atoms, thus the contrast difference between the biological sample and the surrounding resin is minimal. Due to that reason in the last decades, several staining solutions and approaches, performed with heavy metal salts, have been developed with the purpose of enhancing both the intrinsic sample contrast and the differences between the sample and resin. The best staining was achieved with the uranyl acetate (UA) solution, which has been the election method for the study of morphology in biological samples. More recently several alternatives for UA have been proposed to get rid of its radiogenic issues, but to date none of these solutions has achieved efficiencies comparable to UA. In this work, we propose a different staining solution (X Solution or X SOL), characterized by lanthanide polyoxometalates (LnPOMs) as heavy atoms source, which could be used alternatively to UA in negative staining (NS), in en bloc staining, and post sectioning staining (PSS) of biological samples. Furthermore, we show an extensive chemical characterization of the LnPOM species present in the solution and the detailed work for its final formulation, which brought remarkable results, and even better performances than UA.

## Introduction

Transmission Electron Microscopy (TEM) is an established pillar in ultrastructural analysis of biological samples, with countless applications both in research and clinical environment. Biological specimens are formed by light atoms therefore the local tiny density variations should be enhanced to give a detectable contrast in electron micrographs. This is usually achieved by using heavy metals as staining agent^1^. In this field, uranyl acetate (UA) solution has been considered as the gold standard for electron microscopy (EM) characterization, due to its low cost, the very high contrast provided and its intrinsic affinity for biological samples^2^.

Recently, however, the mildly radioactive nature of Uranium-derivatives raised considerable regulatory issues for their use (such as health, availability, price, storage, and disposal). These factors raised an increasing interest in developing safer, more effective alternatives to UA, and several uranium-free staining solutions have been proposed^3–7^.

However, to date, UA is still considered, by electron microscopists working in TEM biology, as the best choice as staining solution.

In the framework of our long-lasting research activity on EM and lanthanide-based chemistry, we sought to develop a novel contrast agent that could provide improved results compared with UA, without its limitation of toxicity and radioactivity.

To this end, among the elements of lanthanide family, we focused on Ytterbium, which shows a very high electron scattering power, because of the contraction of its electronic shell. Lanthanide salts were recently described for their use in TEM^4^, but they usually provide unsatisfactory results due to their low and rather unspecific binding capability. To address this issue, we envisaged a different approach that could combine: (1) typical selectivity of contrast agents, (2) enhanced contrast. This could ultimately lead to a synergistic effect that allows an improved visualization of subtle details usually characterized by very low-contrast.

UA, for example, has been used together with phosphotungstic acid (PTA) exploiting a multiple staining method (MSM) strategy, because the first shows a higher affinity for acid compounds while the last for basic ones. To improve affinity of Yb^3+^ towards biological samples, we decided to investigate its combined use with PTA in an MSM approach.

In the literature, PTA is described as staining agent for TEM^8^, and in particular for negative staining (NS) experiments, although the contrast provided is often not satisfactory, due to the low atomic weight of Tungsten. However, there are several reports of strong interaction between PTA and lanthanide salts^9^, thus we envisioned that an appropriate combination of the two components could merge advantages of both reagents, leading to a more effective staining.

In this work, we describe our results in the rational development of X Solution, an innovative staining agent based on a pH buffered Yb^3+^/PTA mixture, and we show how, under optimized conditions, our staining agent significantly improves upon the staining efficiency of UA, in en bloc staining as well as in NS, and post sectioning staining (PSS) procedures.

## Results

### Chemical characterization

We started our investigation with a study of complexation process between PTA and Yb salts. The aqueous solution chemistry of PTA is remarkably rich, characterized by the presence of different molecular species, originating from phophotungstate [PW_12_O_40_]^3−^ anion by association or loss of fragments (mainly W=O units or tungstate WO_4_^2−^) as a function of pH and/or presence of organic solvents (ethanol, acetone)^10^. Interestingly, phosphorus NMR (^31^P-NMR) (altogether with ^183^W-NMR) has been extensively used to monitor the dissociation of PTA or its complexation with metallic cation in water solution^10,11^. Among others, lanthanide trivalent ions complexation with PTA to form LnPOMs has already been object of different studies, considering the strong oxophilicity of Ln ions and their ability to bind donor atoms following different regular or distorted geometries^12^.

PTA showed its typical ^31^P-NMR spectrum (water/ethanol 80/20 v/v), characterized by the − 15 ppm signal of [PW_12_O_40_]^3−^ specie at low pH (1–3), and by the unique − 10.8 ppm resonance for the monolacunary [PW_11_O_39_]^7−^ specie from slightly acidic to neutral pH (4–7) (Supplementary Fig. S1)^10,11^.

The presence of certain percentage of ethanol has been introduced either to enhance the effectiveness of the staining protocol, considering its beneficial effect for membrane permeation, and also to stabilize PTA species from uncontrolled dissociation^10^.

**Figure 1:**
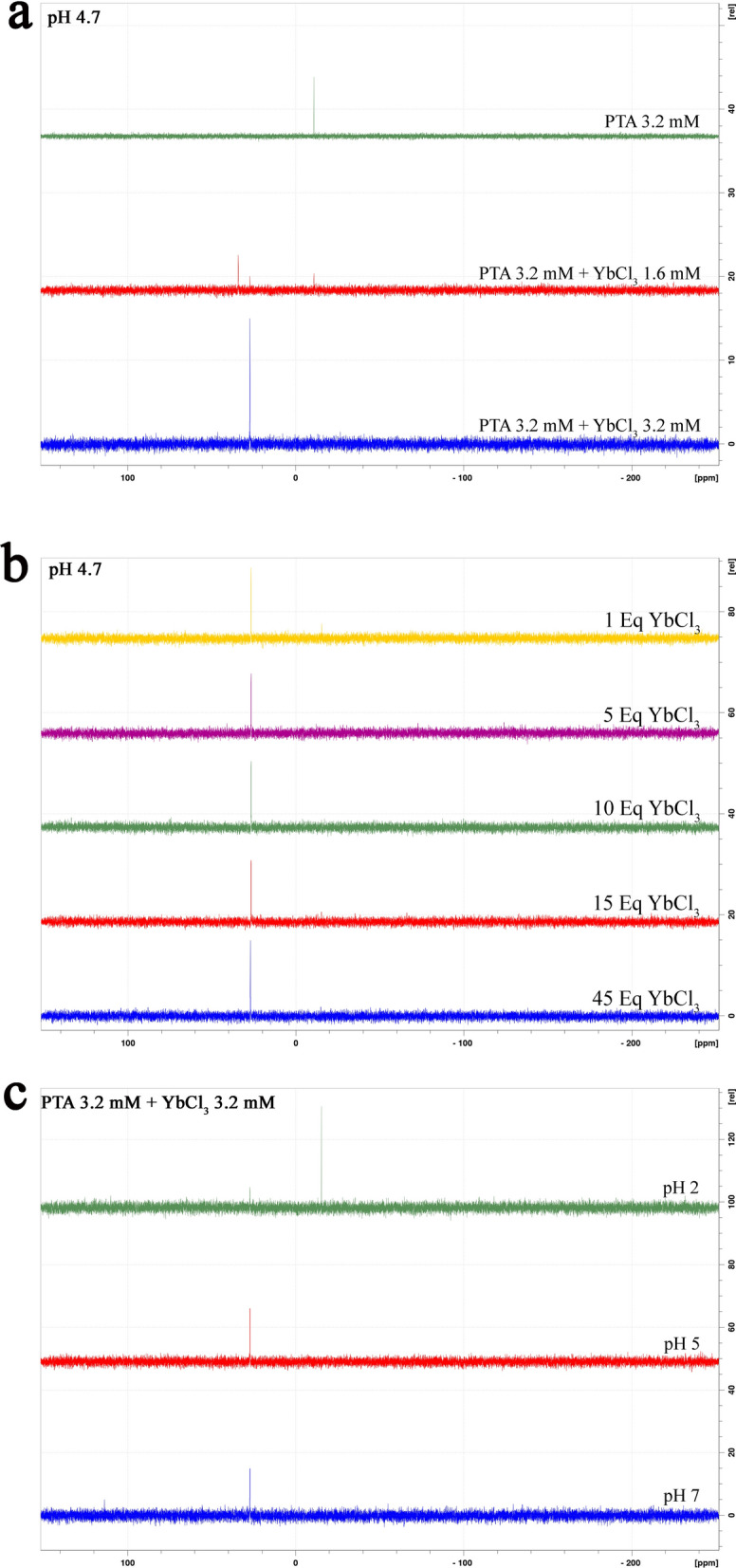
NMR study of complex formation and stability: (**a**) ^31^P-NMR titration of PTA with increasing amounts of YbCl_3_. (**b**) ^31^P-NMR spectra of the active specie in a range of YbCl_3_/PTA molar ratios. (**c**) ^31^P-NMR spectra of a 1:1 mixture of PTA and YbCl_3_ in a pH = 2–7 range.

The PTA complexation with YbCl_3_ salt was followed by ^31^P-NMR (Fig. 1A) and ICP-MS: upon addition of Yb^3+^ salt to a 3.2 mM solution of PTA at pH = 4.7 (water/ethanol 80/20 v/v), we observed the disappearance of the − 10.8 ppm signal of the monolacunary specie, to be replaced after the addition of 1 equivalents of Yb^3+^ with a unique 27 ppm resonance. This 1:1 stoichiometry specie remained unaltered upon addition of an excess of lanthanide, which solely determined the precipitation of a white solid identified as Yb(HWO_4_)_3_ (WO_4_^2−^ pKa_2_ = 3.5)^13^. The 27 ppm resonance is always present in a pH = 2–7 (Fig. 1B, C) value span, even if at low pH altogether with the − 15 ppm uncomplexed [PW_12_O_40_]^3−^ (Fig. 1C). The remarkable chemical shift variation from − 10.8 to 27 ppm, as a result of the complexation, is due to the paramagnetism of Yb^3+^ close in space to the phosphorus atom, and, interestingly, it seemed to be in line with already reported Yb/PTA complexes^14,15^ of different stoichiometry: while in literature are mainly present examples of Yb/PTA 1:2 species with one or two ^31^P-NMR resonances, depending on the symmetry of the system, in our case the 1:1 stoichiometry is unquestionable (with 0.5 equivalents of Yb^3+^ there is also the presence of a 34 ppm resonance of the 1:2 complex) and the identity of the LnPOMs has been undoubtedly confirmed by ICP-MS as the [YbPW_9_O_34_]^6−^ (sodium salt) complex of Yb^3+^ with the trilacunary [PW_9_O_34_]^9−^ species. The reaction in basic condition was not investigated due to the known decomposition of the PTA ensemble and precipitation of the lanthanide oxide/hydroxide.

With a full characterization in our hands, we designed the possible staining solution (X Solution) as a specific LnPOM and we moved deep into its application to biological specimens.

### Method for quantitative analysis

We started our investigation using Mia PaCa-2 cells (en bloc stained and embedded in resin, as described below) and we collected TEM micrographs of Endoplasmic Reticulum (ER) Cisternae, which were located in the cytoplasm of the perinuclear region (10–15 ER images for each experimental point).

First, we established a reliable quantitative protocol to better compare the staining efficiency of the different solutions.

The evaluation of contrast as a normalization of signal to noise ratio (we use S/N in the manuscript referring to these data) was performed as described in the material and method chapter and summarized in the Supplementary Fig. S2.

### Testing the optimal recipe in EM characterization

Firstly, we compared the staining effectiveness of the two components (YbCl_3_ 48 mM or PTA 3.2 mM, both in water/ethanol 80/20 v/v) with X Solution. As already mentioned, both lanthanide salts and PTA have been individually used in TEM, thus a certain degree of staining could be expected^3–8^.

We then evaluated the best molar ratio between YbCl_3_ and PTA in terms of staining efficiency (Supplementary Fig. S3). Only one species containing Phosphorus appears in solution in all YbCl_3_/PTA molar ratios greater than 1 (Fig. 1B). However, we observed a striking dependence of staining efficiency on the excess of YbCl_3_ (Supplementary Fig. S3A, B). Indeed, X Solution shows the best efficiency when YbCl_3_/PTA molar ratio is 15:1, while contrast is poorer than UA for ratios lower than 10:1. Higher ratios than 15:1 lead to salt precipitation and hence increasing the background.

A possible explanation of the contrast increase could be the accumulation of Yb^3+^ ions on a single PTA molecule after the interaction of the latter with the biological specimen. The consequent Yb^3+^ accumulation induces a significant improvement of the contrast.

A confirmation of this accumulation process is provided by the fact that a better contrast is achieved when PTA and YbCl_3_ are added in this order to the specimen rather than if the reagents (i.e. YbCl_3_ and PTA) are added in reverse order. Interestingly the contrast achieved by adding the two reagents one after the other in any of the two combinations is remarkably worse than the one obtained with the preformed X Solution.

Moreover, we observed that X Solution can be kept for a longer time (with respect to the single components) without suffering variation of pH or reduction of staining efficiency. Furthermore, for routinely analysis, the use of a single solution is easier and faster.

**Figure 2:**
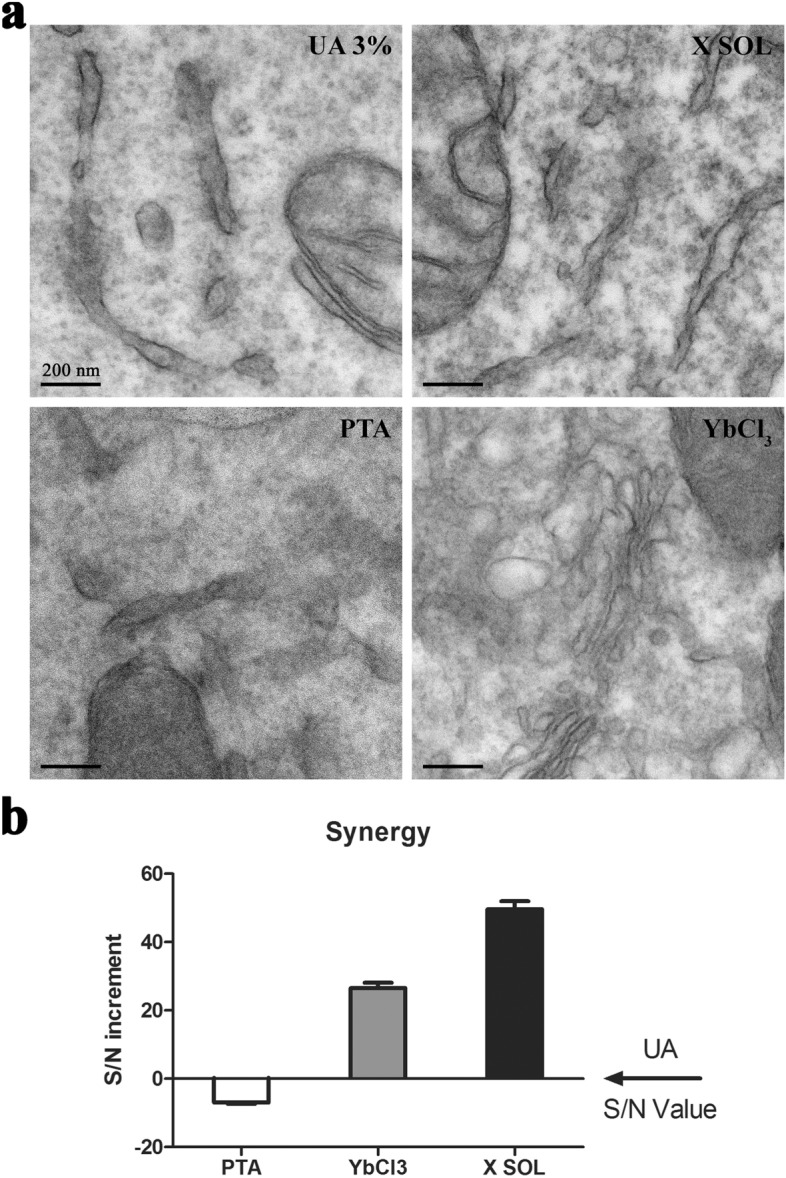
PTA and YbCl_3_ synergy: (**a**) EM micrographs of Mia PaCa-2 cells stained with UA 3% solution, X SOL, PTA 3.2 mM, YbCl_3_ 48 mM (all solutions are made in water/ethanol 80/20 v/v); (**b**) quantitative analysis of the staining efficiency on Mia PaCa-2 cells.

Finally, we observed that increasing the incubation time from 30 min to 1 h leads to improved contrast; this timing was thus chosen as the standard incubation time for all subsequent experiments of en bloc staining during the embedding procedure. The results of the comparison between the staining efficiency of the optimized X Solution (ratio 15:1), PTA 3.2 mM, YbCl_3_ 48 mM alone and UA 3% (all in water/ethanol 80/20 v/v) is summarized in Fig. 2. It is evident that X Solution provides a far better contrast compared with the use of either single components or UA at the same concentration.

In fact, both PTA and YbCl_3_ did not show any concentration-dependent contrast improvement in the 3–5% w/w range (data not shown). The worse performance achieved with single components can be explained by the poor YbCl_3_ selectivity, which leads to unspecific staining, and by poor PTA contrast, due to the relatively low atomic number (i.e. electron density) of tungsten. The combination of the two leads to a synergistic effect that translates in an impressive 48% contrast increase compared with benchmark contrast agent UA 3% (Fig. 2B).

A good contrast in EM is basically due to the combination of selective adsorption and efficient scattering of the electron beam. Uranyl acetate has an intrinsically higher efficiency compared with lanthanides in terms of electrondensity due to its atomic weight and reasonable affinity towards proteins, thus acting both as staining and binding agent. Our X Solution shows a great contrast improvement, due to the enhanced and cleaner interaction with biologic tissue typical of PTA^8,16^, which is also able to localize an Yb deposition around biological features.

We then moved towards a further refinement of the production process of our X Solution. To this end, we tried to address the main potential issue in the routine use of this contrast agent, i.e. pH stability over time. As mentioned, the preparation process of X Solution requires tightly controlled pH conditions. However, an unbuffered system does not guarantee pH stability over the time, and stability studies performed on unbuffered solutions showed a pH decrease of 0.8 units within 13 days. We also demonstrated that such pH variations correspond to variation in the stability of the active compound.

**Figure 3:**
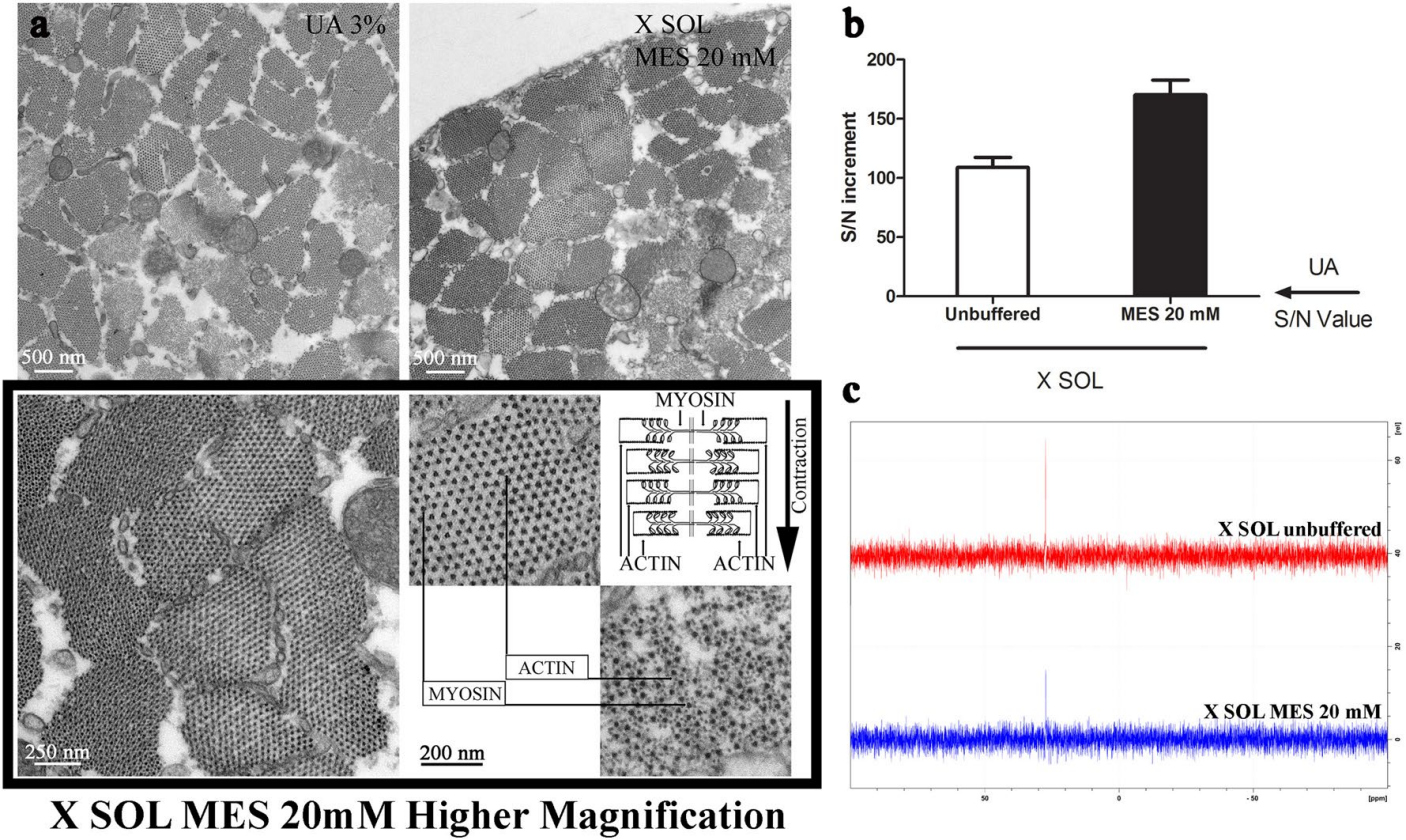
Buffering: (**a**) EM micrographs of gastrocnemius muscle stained with UA 3% solution or X SOL (upper line). The staining seems much more uniform in UA 3% stained samples compared to X SOL stained one; higher magnification of X SOL stained muscle (boxed lower line) shows that differences in contrast is due to the contraction status of myofibers, thus discriminating between relaxed and contracted ones; (**b**) Quantitative analysis of the staining efficiency performed on Mia PaCa-2 cells; (**c**) ^31^P-NMR analysis of X SOL w/wo the addition of MES buffer.

Thus, we screened commonly used buffers (sodium cacodylate, 4-Morpholineethanesulfonic acid (MES), citrate, ammonium acetate) to find the most appropriate to preserve the active species considering their optimal buffering capacity at pH typically used in conventional staining procedures for EM. NMR monitored the effect of adding the buffer to the active species. As shown in Supplementary Fig. S4A, in two cases (sodium Cacodylate and ammonium acetate) the active species was not observed in solution, while MES seems to fully preserve functionality regardless of buffer concentration in the examined range (0–40 mM) (Fig. 3C and Supplementary Fig. S4A).

The same effect on the active species stabilization was observed using sodium citrate, which however does have solubility issues and was not further considered. These findings have an impact on the staining efficiency which is optimal in the case of MES buffered solutions (Fig. 3A, B) and detrimental when the active species is not present (i.e. in solutions buffered with ammonium acetate or sodium cacodylate, Supplementary Fig. S4B). Moreover, our results indicate that X Solution, buffered with 20 mM MES, was stable for not less than one year.

### Non-standard EM application of X Solution

#### X Solution en bloc staining for tissues

When tested for tissue staining (using a gastrocnemius muscle of C57BL6 WT mice at P15, Fig. 3A), our protocol provided an excellent contrast evidencing muscular fibers.

Interestingly, the tissues stained with our X Solution display a not uniform contrast of myofibers, compared with UA stained samples (Fig. 3A upper line). At higher magnification (Fig. 3A lower boxed line), we can confirm that this effect is not due to preparation artifacts, but rather to an increased capability of X Solution to highlight light structures such as actin chains^17^, allowing better discerning contraction state of muscular fibers.

This in turn would allow a more straightforward evaluation of the final image, ultimately leading to easier evaluation either by operator or by automated image analysis protocols, and hence to faster processing of image outputs.

#### X Solution negative staining (NS) and dilutions

**Figure 4:**
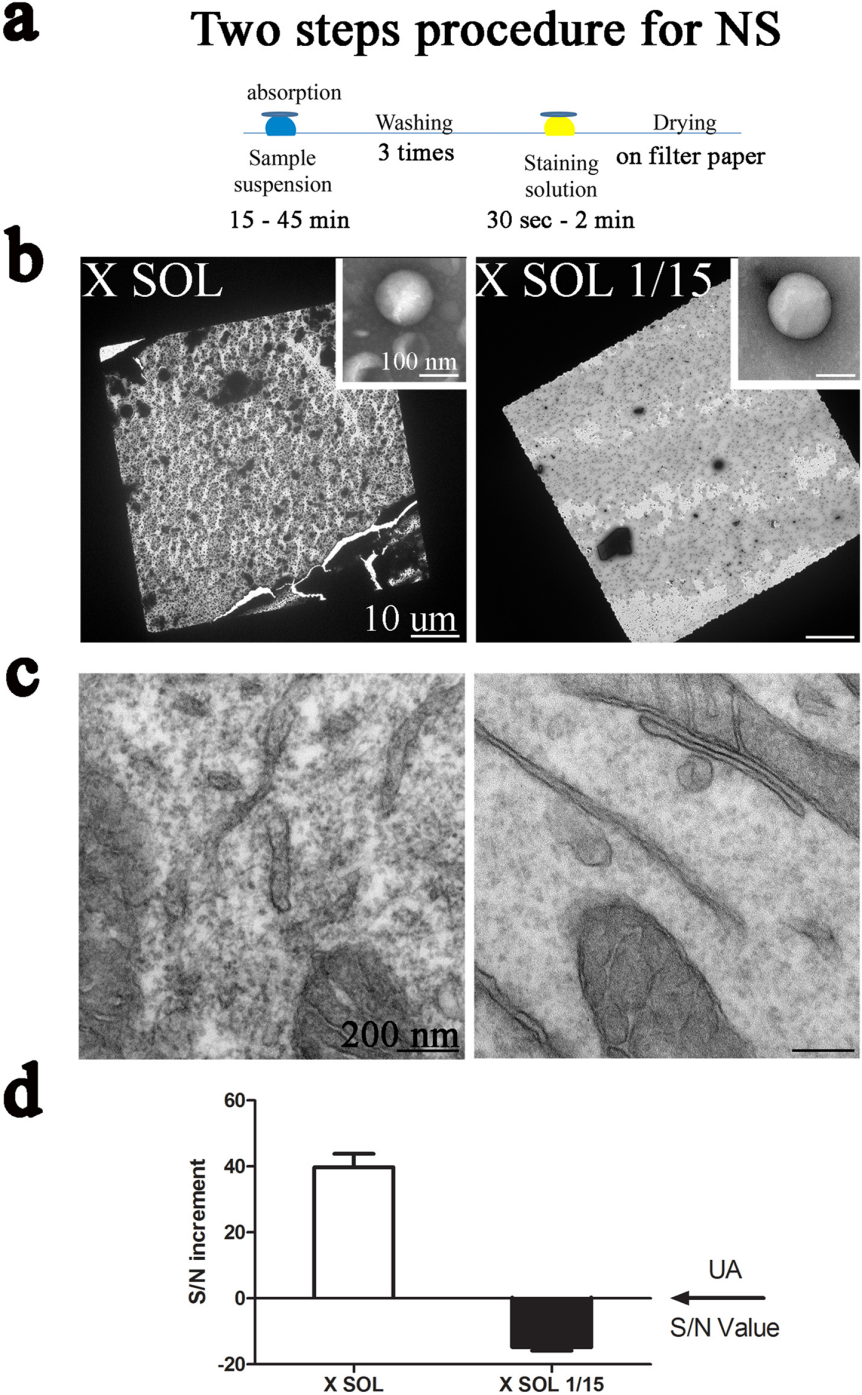
NS and dilution: (**a**) representative scheme of the two steps protocol for NS; (**b**) EM micrographs of synthetic liposomes negatively stained with X Solution, pure or diluted in water 1/15 (V/V); (**c**) EM micrographs of Mia PaCa-2 cells en bloc stained with X Solution, pure or diluted in water 1/15 (V/V); (**d**) Quantitative analysis of the staining efficiency performed on Mia PaCa-2 cells.

Negative Staining is an approach conventionally applied to suspended samples, for example viruses, liposomes, exosomes etc. In Fig. 4A and Supplementary Fig. S5A, a conventional two-steps protocol for NS is reported. As reference sample we selected a suspension of synthetic liposomes^18^ that were stained with the pure or the diluted solution 1/15 in water (V/V—Fig. 4B).

Our solution is equally effective in NS, with better results obtained at relatively high dilutions (Fig. 4B and Supplementary Fig. S5C). Indeed, the dilution in water allows lowering the percentage of ethanol of the staining solution. In this way, we were able to ultimately reduce the shrinkage effect usually observed when hydro-alcoholic solutions for NS are used. The active species remains stable (as observed by NMR) even at 15/1 dilutions (Supplementary Fig. S5B).

When the diluted solution is used for the en bloc staining of Mia PaCa-2 cells, a reasonable staining can be observed, albeit with a lower contrast compared with the concentrated solution (shown in Fig. 4C and quantified in Fig. 4D). In a future perspective, the possibility to achieve good contrast even at high dilutions can open the way to the effective and fast staining of delicate biological structures, such as primary cultures of neurons, even if in these cases the solution should maintain the same water to ethanol ratio.

#### X Solution post-sectioning staining (PSS)

Post-sectioning staining was performed on Mia PaCa-2 cells embedded with a chemical protocol that excludes the staining step. Briefly, after the sectioning, grids were incubated with the staining solution for different times (Supplementary Fig. S6A).

In Supplementary Fig. S6B (upper line), the efficiency of the staining could be easily evaluated considering two parameters: (1) the contrast of ER cisternae, (2) the darkness of the background (cytoplasm). We observed regular increase of the contrast with the staining time reaching a maximum for 15 min of treatment. For longer time the background become dominant and hence contrast decreases. Note that the same procedure applied to en bloc stained samples does not produce any contrast increase (Supplementary Fig. S6B lower line).

#### X Solution affinity for organic phosphates: possible mechanism

Finally, we performed an additional comparative investigation exploiting both NMR and TEM analysis to gain an insight in the working principles behind the efficiency of our X Solution. A closer look at TEM images suggests that phosphate groups could be important players in the binding process of PTA/YbCl_3_ to biological structures.

**Figure 5:**
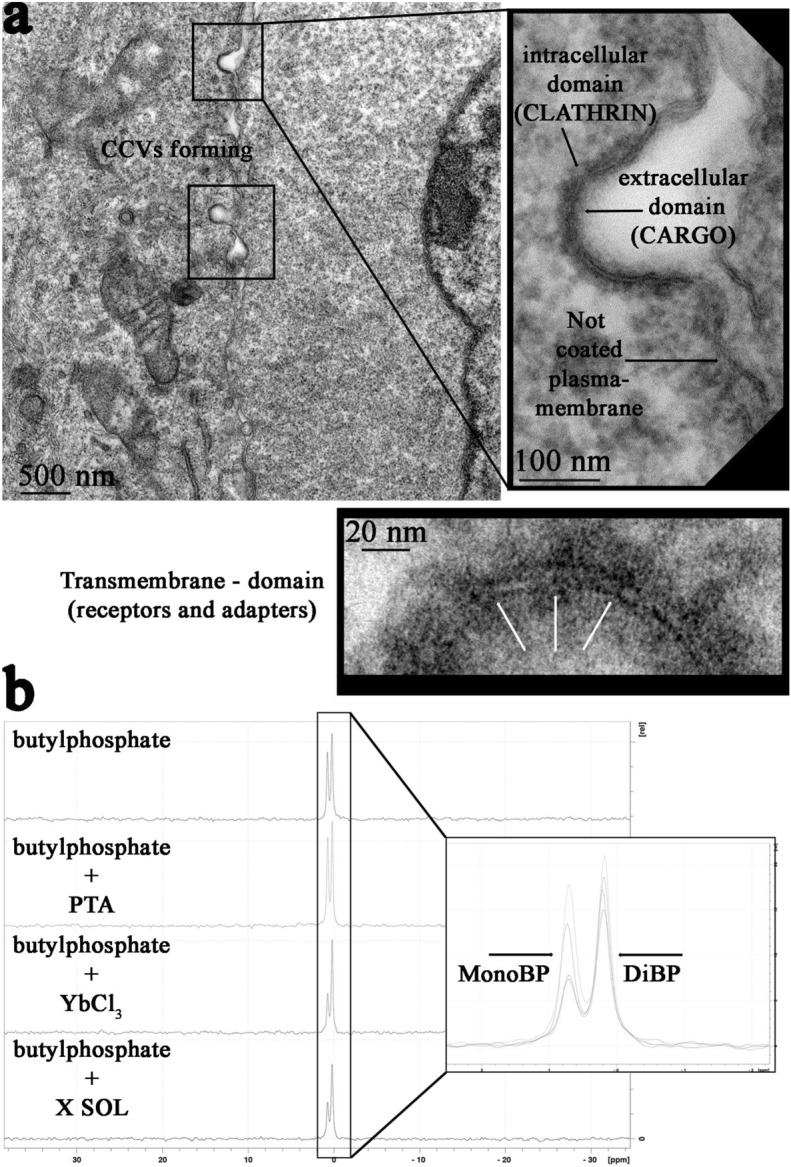
Mechanistic hypothesis of X Solution staining process: (**a**) Clathrin coated vesicles formation in embedded Mia PaCa-2 cells en bloc stained with X SOL; X Solution clearly shows clathrin shells in the cytosolic side, cargo protein in the extracellular side, and, also, proteins’ transmembrane domains (white arrows); (**b**) ^31^P-NMR of the interaction between a mixed solution of mono-and dibuthylphosphate: alone or associated with PTA, YbCl_3_ and X Solution.

To address this issue, we chose clathrin mediated endocytosis as a possible representative process able to clarify the interaction between our staining solution and biological samples (Fig. 5).

Endocytosis is the primary mechanism trough which eukaryotic cells are able to internalize and deliver cargos into the cell^19^, but it is also a clear example of both protein–protein and protein–phospholipids interaction^20^.

During this process phospholipids and phosphorylated proteins, as well as energy molecules (ATP and GTP), are enriched in the region corresponding to endocytosis mechanism. We could distinguish three main domains of interest in a forming clathrin coated pit (highlighted in Fig. 5A): (1) intracellular domain, characterized by clathrin triskelia and their adapters; (2) extracellular domain, that mediates the interaction between cargo and binding proteins, which is enriched of GDP molecules; (3) transmembrane domain comprising the transmembrane regions of membrane proteins (white arrows). In cells stained with X Solution we observed a very strong definition of these clathrin coated structures (CCS) with respect to the neighboring regions not involved in endocytosis. Moreover, we noted that the remarkable contrast we obtained with our staining led us to the unequivocal identification of the three mentioned domains. In our hands, this might suggest that the enrichment in phosphate groups around CCS, is the vehicle for a proper staining through the interaction either with PTA or YbCl_3_.

To validate this mechanistic hypothesis, we investigated by ^31^P-NMR the interaction of our solution in the presence of a circa 1:1 mixture of mono- and dibutylphosphate (Fig. 5B), where the former mimics a phosphorylated protein. We found that there is a clear influence of lanthanide salt specifically on the signal of monobutylphosphate, which broadens upon addition of the lanthanide. This effect can be attributed to the interaction of the monoester phosphate with the Yb paramagnetic ion accompanied by a partial precipitation of complexed monobutylphosphate. This could explain the observed efficient staining of phosphorylated proteins in TEM. Note that a minor but not negligible contribution must arise from phosphate head groups in lipids.

## Conclusion

In conclusion, we developed and validated a new contrast agent for TEM of biological samples based on lanthanide polyoxometalates. This staining agent outperforms other uranium-free staining solutions and favorably compares even with gold standard uranyl acetate (Supplementary Fig. S7). The observed contrast improvement, that can reach almost 50% compared with uranyl acetate, allows improved evaluation of biologic details, and could ease, in perspective, automated pattern recognition by image processing algorithms. Finally, the absence of radioactive material in our contrast agent makes it sensibly less toxic compared with the mildly radioactive uranium acetate. Thus, its use allows overcoming current complications and cost increases due to acquisition, handling, and disposal of radioactive substances, ultimately making X solution more attractive also from a cost/effectiveness perspective.

## Material and methods

### Chemical characterization

All the chemicals for synthesis and characterization of X Solution were purchased from Sigma-Aldrich, with exception of Nitric acid for trace analysis, which was purchased from Merck.

All ^31^P-NMR spectra were obtained with a Bruker ULTRASHIELD 300 MHz. Delay time (d1) was selected at 2.000 s and the Pulse (p1) was selected at 11.00 µs. The number of scans was set to 100 scans. The inductively coupled plasma-mass spectrometry (ICP-MS) analysis was performed with an Agilent Technologies 7700 Series ICP-MS.

#### Solution preparation

In a typical experiment, 92 mg of Phosphotungstic acid dissolved in 3.5 mL of H_2_O was partially neutralized with 1 M NaOH up to pH 4–5. Then, 186 mg of YbCl_3_ dissolved in 4 mL of H_2_O were added and the solution volume was brought to 8 mL with H_2_O. Finally, 2 mL of absolute ethanol were added, and the resulting suspension was stirred for 24 h at room temperature. Precipitate was removed by centrifugation and MES (39 mg) was added. pH was adjusted to 4.6–4.8 and the suspension was further stirred for 24 h. Finally, suspension was filtered with a 0.22 µm filter, and the solution was stored in the dark at 4 °C.

#### NMR

##### Stoichiometry evaluation

Two solutions were prepared separately: (1) PTA stock solution in MilliQ/Ethanol (EtOH) (80:20 v/v); (2) YbCl_3_ stock solution in MilliQ H_2_O/EtOH (20% v/v). The pH of these solutions was adjusted to 4.7 with NaOH (0.1 M). The final concentration of the stock solution was 6.4 mM and 96.0 mM for PTA and YbCl_3_ respectively.

Control sample (PTA alone) is formed from 250 µL of the PTA stock solution and 250 µL of EtOH (20%). To obtain the YbCl_3_/PTA 0.5 equivalent solution, 250.0 µL of the PTA solution were mixed with 8.3 µL of the YbCl_3_ solution and 241.7 µL of EtOH (20%). The YbCl_3_/PTA 1.0 solution was obtained mixing 250.0 µL of the PTA solution with 16.6 µL of the YbCl_3_ solution and 233.4 µL of EtOH (20%). To every sample were added 10 µL of Deuterium oxide (D_2_O). All the measured ratios were obtained by changing the volume of YbCl_3_ solution added and maintaining unaltered the volume of the PTA solution. In this experiment, the number of scans was increased at 500.

##### Titration experiment

PTA was completely dissolved in MilliQ water, desired pH was reached with NaOH 1 M. Finally, the solution was brought to volume to obtain a final concentration of 6.4 mM. For YbCl_3_ a solution, with same parameters of pH and concentration, has been prepared. The samples were obtained mixing 250 µL of the PTA solution with 250 µL of the YbCl_3_ solution and 10 µL of D_2_O. In this experiment, the number of scans was increased at 500.

##### Buffer experiment

For the unbuffered system two different aqueous stock solutions were prepared: (1) PTA (6.4 mM), (2) YbCl_3_ (96.0 mM). The pH of both solutions was adjusted with NaOH (1 M) at pH of 4.7.

250 µL of each solution were mixed 10 µL of D_2_O has been added. Comparison with different buffering system (i.e. ammonium acetate, sodium citrate, sodium cacodylate and MES) was performed adding 20 mM of each buffer to the unbuffered solution.

##### Binding affinity with mono: and dibuthyl phosphate

3 mg of the mixture of mono—and dibuthyl phosphate (BP) were dissolved in 500 µL MES buffer (20 mM) at pH 5, to this solution were added 20 µL D_2_O and 15 µL di MilliQ water/EtOH (80:20 v/v). In the reference spectrum (BP alone) were added other 15 µL of EtOH (20%).

PTA and YbCl_3_ solutions contained, instead of 15 µL of EtOH (20%), 15 µL of the corresponding stock solutions and for X solution 15 µL of our staining solution.

#### ICP-MS and digestion

In a typical experiment the solution dialyzed three times using membranes (MW cut-off 100–500 Da, spectrum lab) against deionized water (1/1,000 volume ratio). The resulting solution was diluted at different ratios (1/10, 1/100 and 1/1,000 v/v) in aqueous HNO_3_ (5%), and the resulting solution was analysed for Yb and P content in ICP-MS comparing the results with a standard curve.

Tungsten content was determined by diluting the solution at the same ratios in aqueous ammonia (5%), and comparing the results with a standard curve. In each case, the derived concentration for each dilution was averaged. The results were compatible with [YbPW_9_O_34_]^6−^ and Yb_2_(HWO_4_)_3_ for the species in solution and the precipitate, respectively.

### Biological samples

#### Liposomes

The appropriate mixture of lipid (Chol/DPPC/Lipopeptide-Stealth, 20:40:40) were resuspended in dimethyl sulfoxide (1 mL of DMSO for 200 µg of total lipids/lipopeptide). The solution was frozen in liquid nitrogen and then freeze-dried. The resulting solid could be stored at − 20 °C for several months without significant alterations. The solid was resuspended in PBS solution and extruded 21 times through a polycarbonate membrane (pore size: 100 nm), keeping the solution at 60 °C with a heated liposomal extruder. This solution can be stored at 4 °C for some weeks.

#### Cell cultures

Human adenocarcinoma pancreatic cells (Mia PaCa-2 cells) were purchased from the American Type Culture Collection (ATCC) and were maintained in Dulbecco’s modified Eagle medium (DMEM) from Invitrogen (Carlsbad, CA). Growth medium was supplemented with 10% fetal bovine serum (FBS), 4 mM l-glutamine, 1 mM sodium pyruvate, 100 U/ml penicillin, and 100 mg/mL streptomycin (Invitrogen). Cells were maintained at 37 °C in a humidified 5% CO_2_ atmosphere.

#### Tissue

Archived tissues used in this project were from previous studies (Permit Number: 0004419)^21^. They were stored in laboratory at 4 °C in Sodium Azide solution. No new animal has been sacrificed for the current study.

Skeletal muscles arising to C57BL6 mice WT P15 have been rinsed to remove the 0.4 M Na Azide.

Then a single gastrocnemius muscle has been dissected in small cubes and used for the test on “more complex samples”.

#### Resin embedding and en bloc staining

Mia PaCa-2 cells were seeded 24 h before each experiment in p30 Petri dish to reach 80–90% of confluence.

The embedding procedure has been performed as described elsewhere^22,23^, except than for the staining step.

Briefly Mia PaCa-2 cells were fixed with 1.5% Glutaraldehyde in sodium cacodylate buffer (0.1 M; pH 7.4), as monolayer, for 1 h at room temperature. Next, cells were manually scraped, collected and centrifuged for 15 min in the same fixative solution until a visible pellet was obtained, and kept in new fixative solution overnight at 4 °C. Cells were then post-fixed with reduced osmium tetroxide (1% OsO_4_ and 1% K_3_Fe(CN)_6_ in sodium cacodylate buffer (0.1 M; pH 7.4). Samples were then rinsed, en bloc stained, for 30 min or 1 h, with different staining solutions. After the dehydration, with a growing series of ethanol concentration, cells were finally embedded in epoxy resin (Epon 812, Electron Microscopy Science, Hatfield, PA, USA) that was then baked for 48 h at 60 °C.

Muscles were rinsed in sodium cacodylate buffer and further dissected into smaller blocks (1 mm^3^) that were subsequently processed for TEM^24^. For these samples we used a fixative solution with a higher concentration of Glutaraldehyde (2% instead of 1.5%) in sodium cacodylate buffer 0.1 M as we usually do for tissue processing in our laboratory. The procedure of embedding after the fixation is the same, we used for Mia PaCa-2 cells.

#### Sectioning

Semithin sections (500 nm) were cut using an ultramicrotome (UC7—Leica Microsystems, Vienna, Austria), were collected on glass coverslips and stained with 0.1% ethylene blue, 0.1% toluidine blue in PB. Samples were analyzed with an optical microscope (DM750, Leica Microsystem, Vienna, Austria), equipped with an ICC50HD (Leica Microsystem, Vienna, Austria) digital camera.

For the ultrastructural analysis, thin 90-nm sections were collected on 300 mesh copper grids.

#### Negative staining (NS)

A two-step protocol was used for the specimen preparation^25^ (Fig. 4A and Supplementary Fig. S5A): liposomes suspensions were adsorbed for 30 min onto carbon-coated 300 mesh copper grids (Electron Microscope Science, Hatfield, PA, USA) washed three times with pure water and stained for 30 s or 1 min with X sol or UA 3% solution, then grids were paper-drained and directly analyzed with a Zeiss Libra 120 Plus transmission electron microscope, operating at 120 kV and equipped with an in-column omega filter and 16-bit CCD camera 2 k × 2 k bottom mounted (Zeiss, Oberkichen, Germany).

#### Post-sectioning staining

Unstained samples were sectioned as previously described and sections on grids were then stained with X Solution for 10, 15 or 20 min. Finally, grids were washed in water bath, drained and imaged.

We have also tested the capability of X Solution to increase the contrast in samples that were already en bloc stained during the embedding protocol.

#### Ultrastructural characterization of embedded samples: qualitative analysis

Different magnification [between 250× (Low Magnification (LM)] to 40.000×) micrographs were collected with the omega filter and used for the first screening of cells contrast to the electron beam. All the parameters for images acquisition were not changed during a single experiment to avoid any variation due to the acquisition itself.

#### Contrast evaluation

The evaluation of contrast was performed on 10–15 images arising from different cells for every experimental point. Micrographs of ER cisternae were all collected at the same magnification (16.000×) maintaining the same acquisition parameters (i.e. brightness, exposure time) for each sample.

Using FIJI software, we evaluated the intensity plot along a line (100-pixel length − 30-pixel width) crossing a single ER cisterna, whit the caution of centering the line at half the diameter of every cisterna. Moreover, at least one end of the line was placed in a clear, cytoplasm region. We used as R value the ratio between the highest and the lowest contrast value for each plot. Contrast values (S/N) were calculated accordingly to the following formula: (R_img_ − 1)/(R_stand_ − 1) × 100, where R_img_ is the ratio for the examined image, and R_stand_ is the ratio for the standard sample used as control for each experiment. R_stand_ used in calculations indicates R of samples used as controls for normalization. Samples stained with UA were used as control (R_stand_) in all experiments.

## Supplementary information

Supplementary information. (PDF 2121 kb)
