# Supplementary material for: Uranium-free X solution: a new generation contrast agent for biological samples ultrastructure
Source: Sci Rep. 2020 Jul 14;10:11540. doi: 10.1038/s41598-020-68405-4 (PMC7360580; doi:10.1038/s41598-020-68405-4)
Supplement: Supplementary file 1 — Supplementary information. (PDF 2121 kb) [file 41598_2020_68405_MOESM1_ESM.pdf]

Uranium-free X solution: a new generation contrast agent for biological samples ultrastructure

Aldo Moscardini[1*], Sebastiano Di Pietro[2*], Giovanni Signore[3§], Paola Parlanti[4], Melissa Santi[5], Mauro Gemmi[5] and Valentina Cappello[5§]
[1] NEST, Scuola Normale Superiore, Piazza San Silvestro 12, 56127, Pisa, Italy
[2] Dipartimento di Frmacia Università degli Studi di Pisa, via Bonanno Pisano 6, 56126 Pisa, Italy
[3] Fondazione Pisana per la Scienza, via F.Giovannini 13, 56017, San Giuliano Terme (PI), Italy
4 Child Health Institute of New Jersey and Department of Neuroscience and Cell Biology, Rutgers Robert Wood Johnson Medical School, 89 French Street, New Brunswick, NJ 08901 USA.
[5] Istituto Italiano di Tecnologia, Center for Nanotechnology Innovation @NEST, Piazza San Silvestro 12, 56127 Pisa, Italy.
[*]those two authors equally contributed to the manuscript
[§] Corresponding authors; Corresponding authors e-mail: 1) TEM and biological samples: valentina.cappello@iit.it; 2) Chemistry and chemical characterization: g.signore@fpscience.it

SUPPORTING INFORMATION

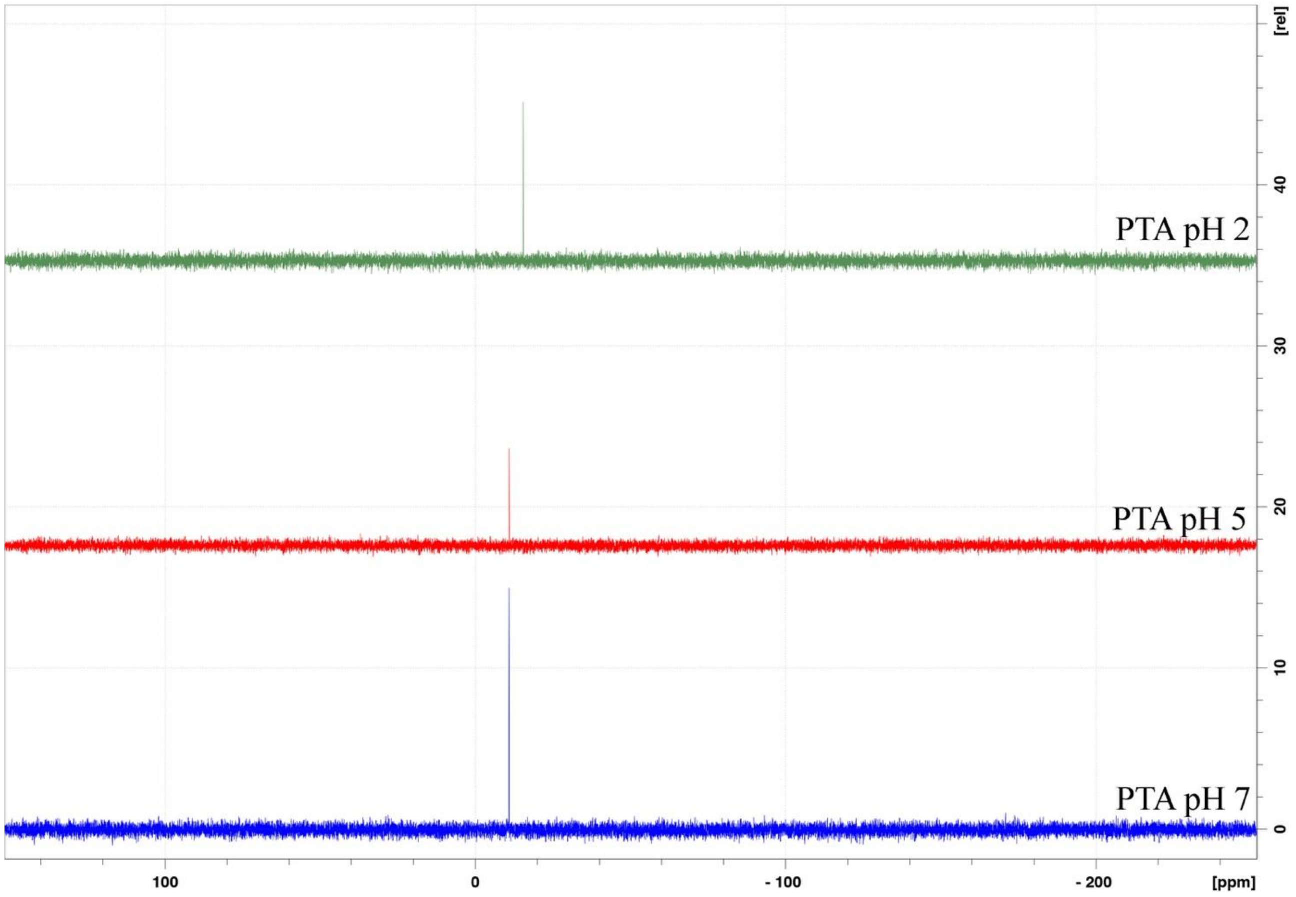

FIGURE S1 NMR study of PTA pH-stability
Speciation of PTA monitored by ^{31}P-NMR in function of pH.

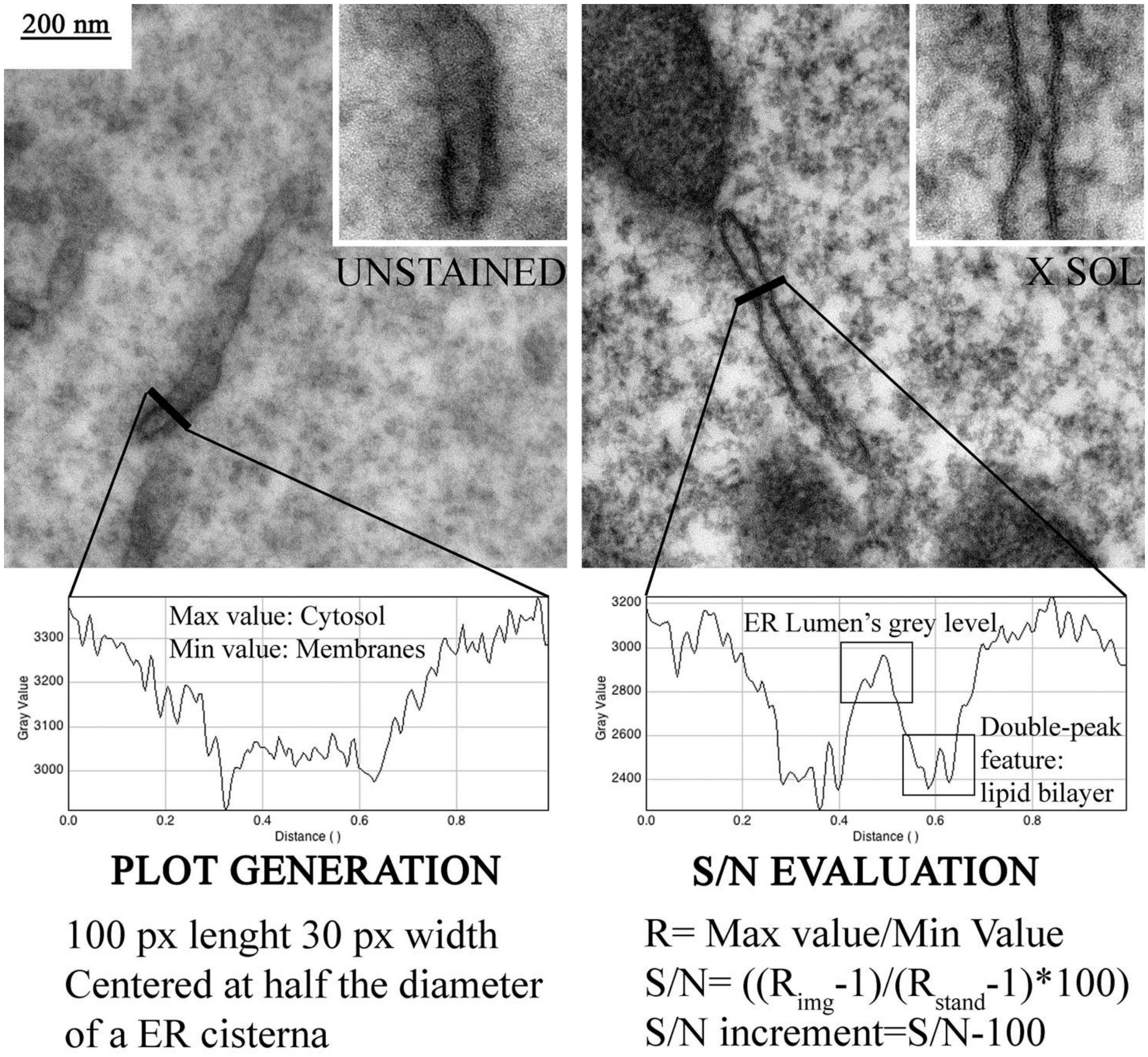

FIGURE S2

Method for the contrast quantitation on ER cisterns:

Plot generation, determination of maximum and minimum values, evaluation of S/N and its increments.

Representative images of Mia PaCa-2 cells unstained or stained with X Solution during the embedding procedure.

R_{stand} used in calculations indicates R of samples used as controls for normalization. Note that unstained samples are shown here only to better clarify the procedure. In all experiments we set samples stained with UA 3% as control (R_{stand})

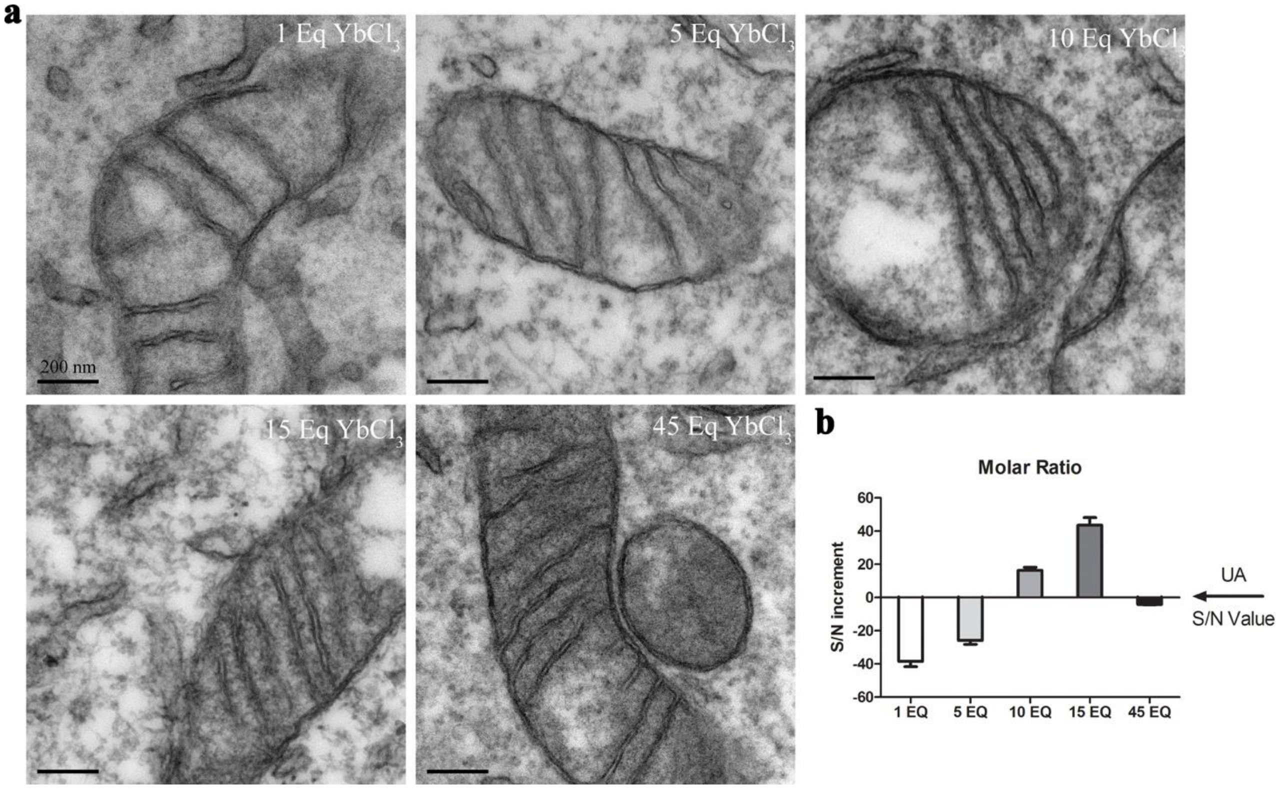

FIGURE S3

Molar Ratio

a) Representative images of mitochondria in embedded Mia PaCa-2 cells: the staining solutions were prepared with a different $YbCl_3$/PTA molar ratio;
b) Quantitative analysis of the staining efficiency

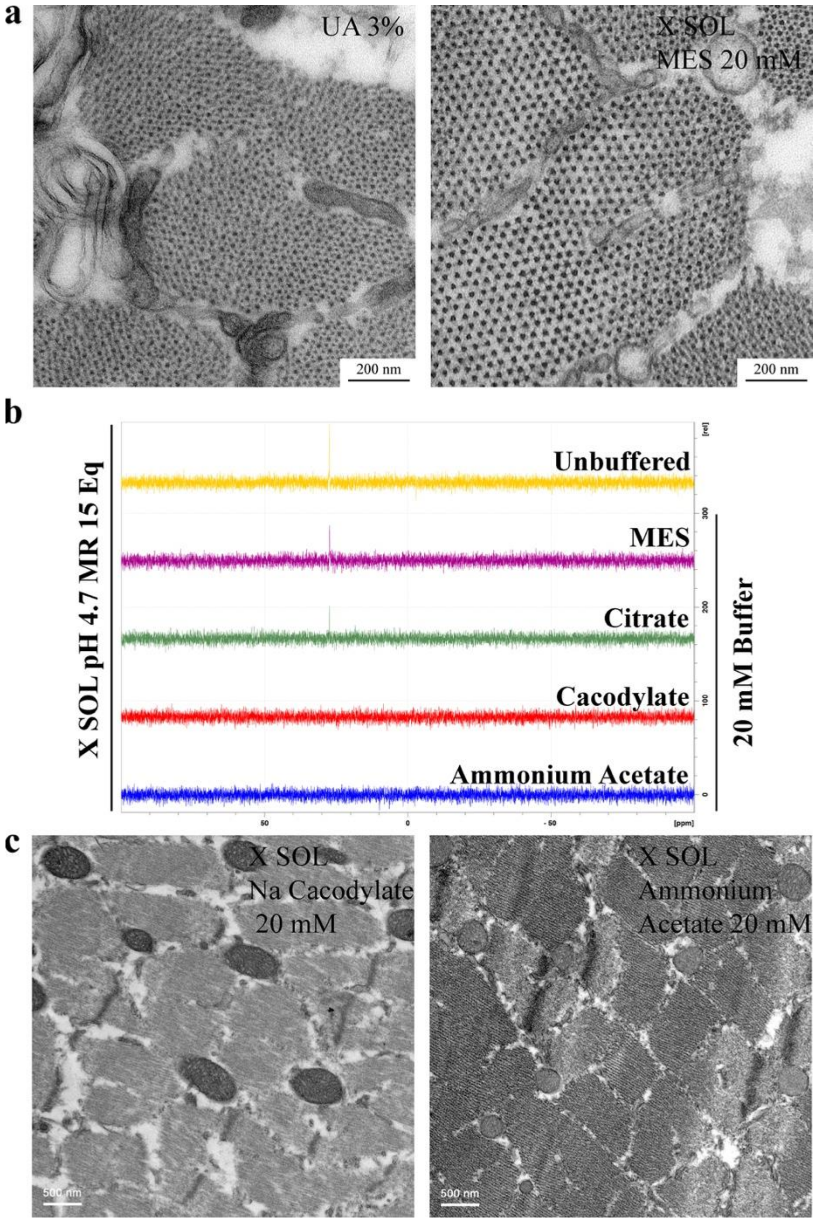

FIGURE S 4

BUFFERING and complex stability

a) EM micrographs of gastrocnemius muscle stained with UA 3% (left panel) or X SOL in MES buffer (right panel);
b) ^{31}P analysis of X solution unbuffered or buffered with different buffers: MES, citrate, sodium cacodylate, ammonium acetate;
c) EM micrographs of gastrocnemius muscle stained with X solution in sodium cacodylate buffer (left panel), X Solution in Ammonium Acetate buffer (right panel).

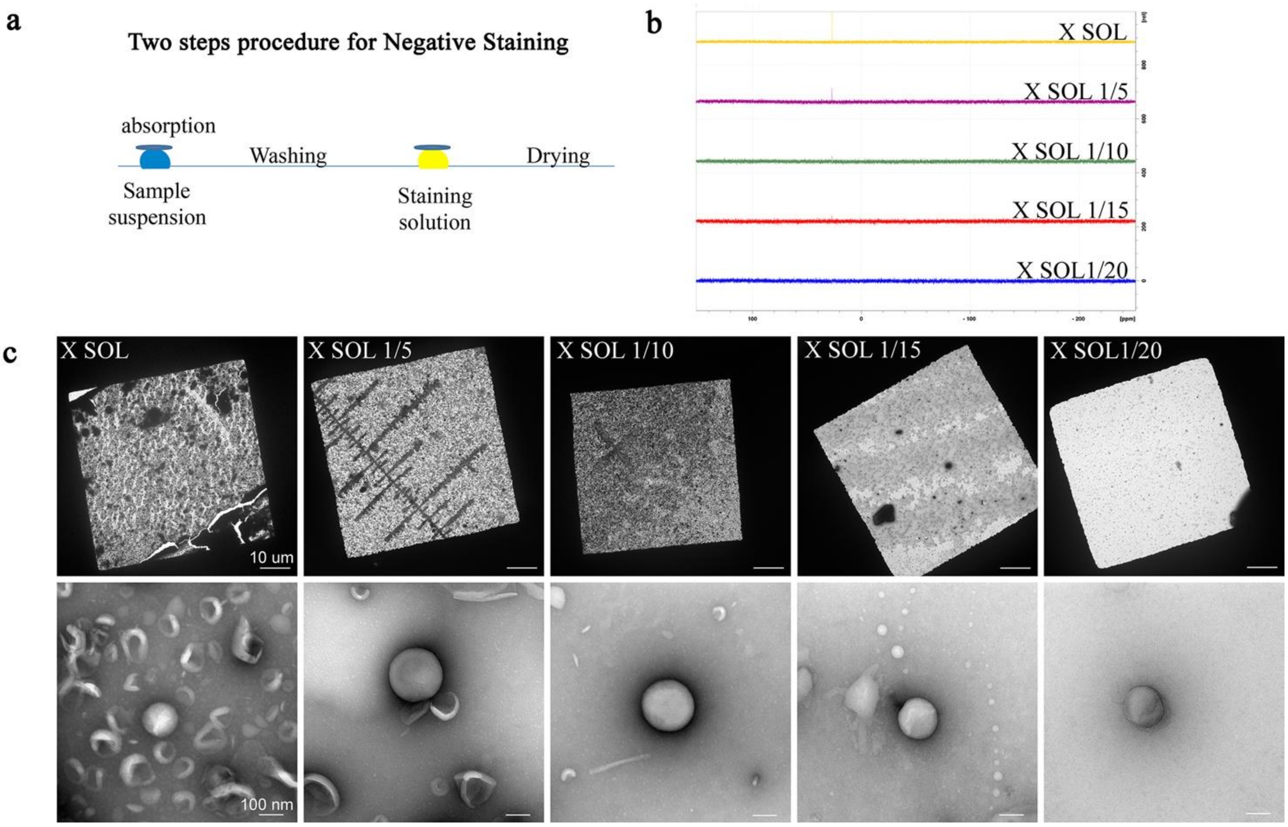

FIGURE S5

Negative staining with different concentration of X SOL

a) Representative scheme of the two steps protocol for NS;
b) ^{31}P-NMR analysis of X solution pure or diluted in EtOH (20%) 5, 10, 15 or 20 times;
c) EM micrographs of synthetic liposomes Negatively stained with X Solution, pure or diluted in water 1/5, 1/10, 1/15 or 1/20 (v/v).

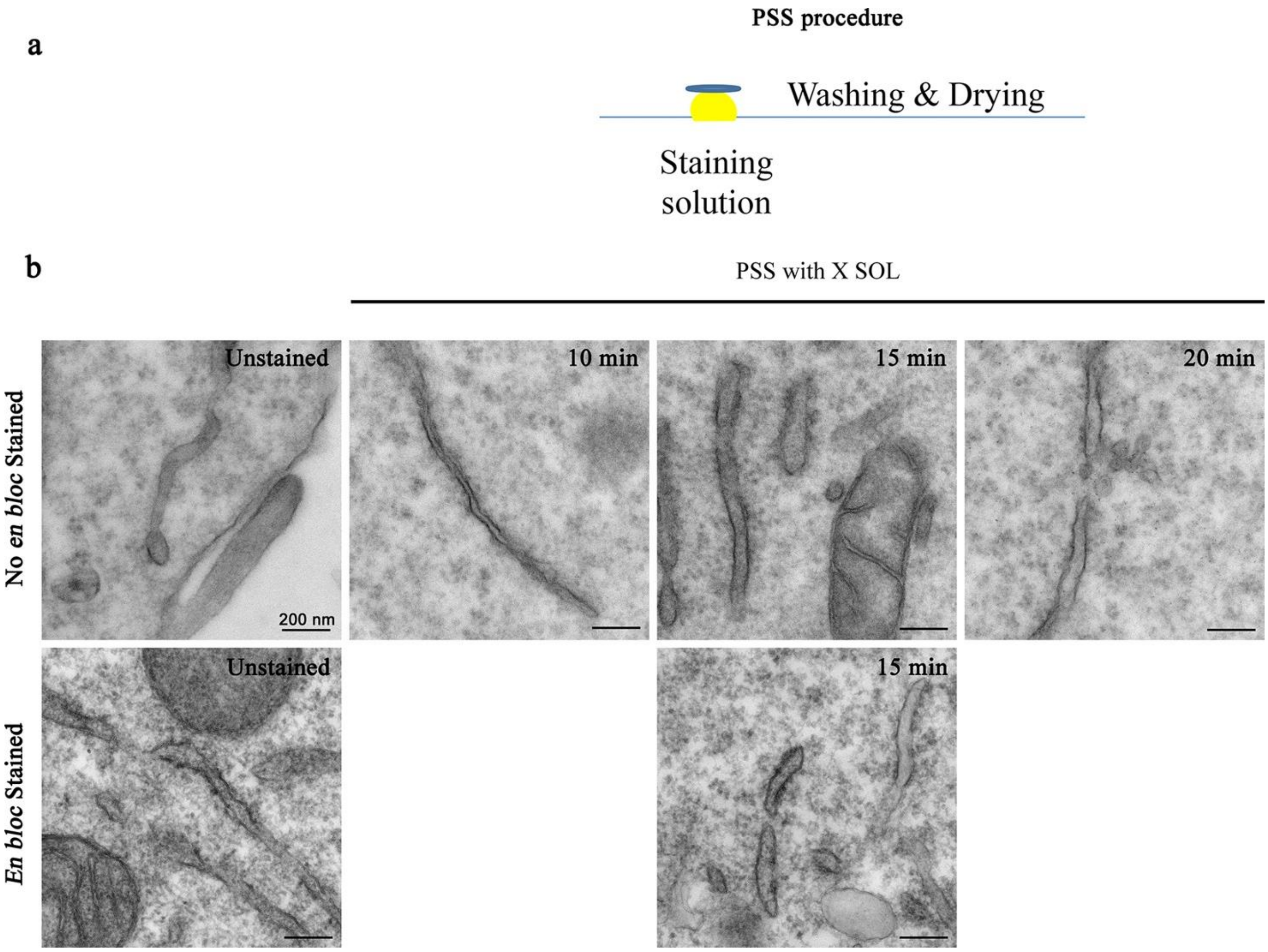

FIGURE S6

Post sectioning staining (PSS) on thin sections

a) Representative scheme of PSS procedure
b) EM micrographs of Mia PaCa-2 cells (upper line: no *en bloc* stained cells. Sections were not stained, or post sectioning stained for 10, 15 or 20 minutes. Bottom line: *en bloc* stained cells. Sections were not stained, or post sectioning stained 15 minutes.

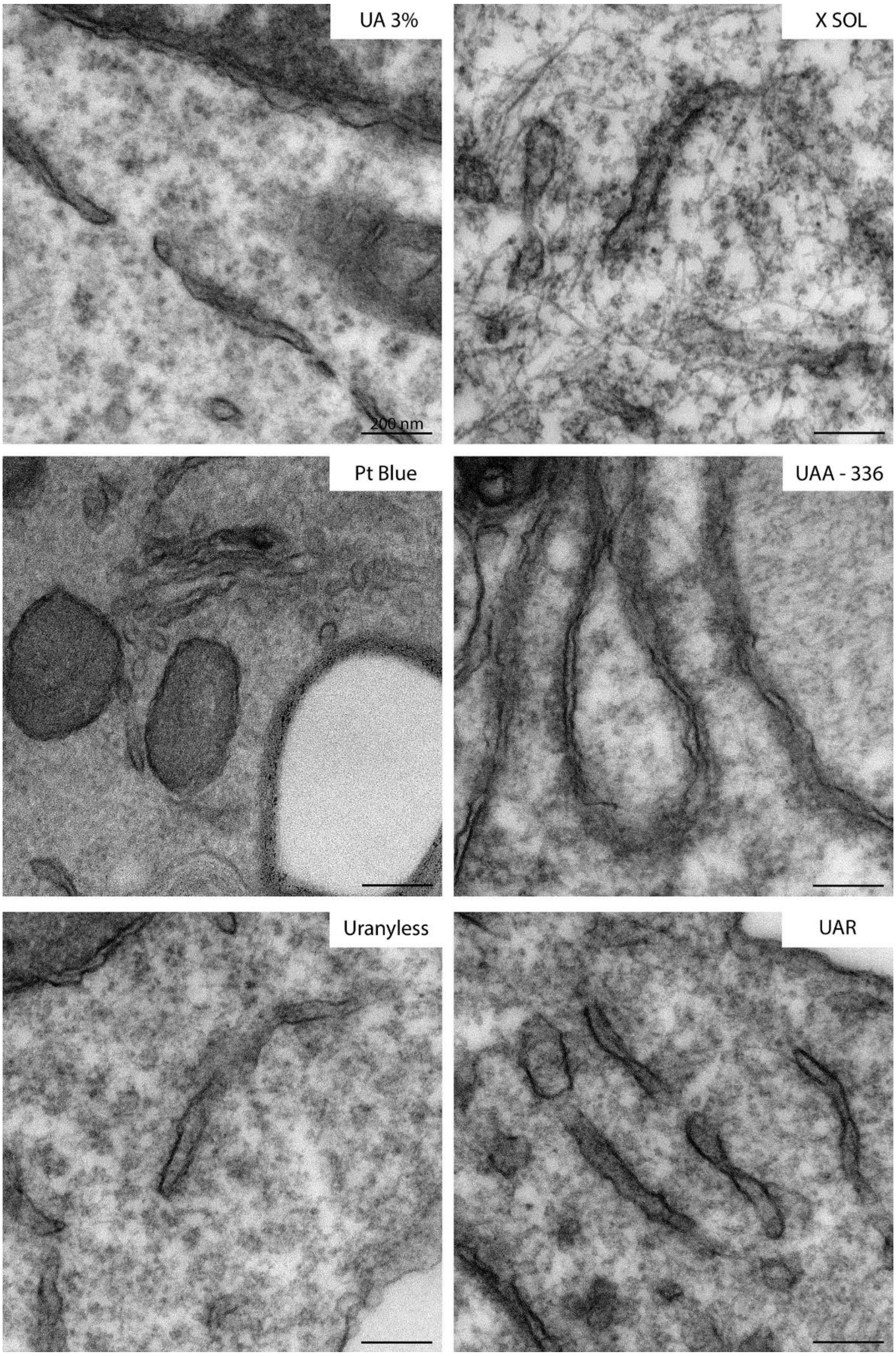

Figure S7

Comparison with Uranium-free commercial alternatives

EM representative images of Mia PaCa-2 cells embedded with the described protocol and stained for one hour with UA, X SOL, Pt Blue (Agar Scientific), Uranyl acetate alternative 336 (Agar Scientific), Uranyless (EMS), and UAR (EMS) respectively.

Tab S1. Quantitave analysis data

Sinergy						
	PTA	YbCl3	X SOL	UA		
Number of v	10	10	10	6		
R	1,133	1,181	1,214	1,143		
Std. Deviatio	0,02534	0,0304	0,01902	0,035		
Relative Erro	0,0223654	0,0257409	0,01566722	0,03062117		
S/N	-6,993007	26,5734266	49,6503497			
Absolute Erro	-0,3705355	1,49773333	2,29823466			
Buffering						
	Unbuffered	MES 20	UA			
Number of v	10	10	10			
R	1,31717415	1,41034399	1,15186872			
Std. Deviatio	0,07006585	0,06713285	0,02975237			
Relative Erro	0,05319406	0,04760034	0,02582966			
S/N	108,847584	170,196518				
Absolute Erro	8,60154095	12,4975292				
Molar Ratio						
	1 EQ	5 Eq	10 EQ	15 EQ	45 EQ	UA
Number of v	10	10	10	10	10	10
R	1,1933669	1,23282491	1,36576954	1,45140042	1,30103385	1,31418227
Std. Deviatio	0,03938965	0,04718535	0,08575106	0,07708648	0,03032194	0,06608294
Relative Erro	0,03300716	0,03827417	0,06278589	0,0531118	0,02330604	0,05028446
S/N	-38,453911	-25,894955	16,4195351	43,6746959	-4,1849661	
Absolute Erro	-3,2028884	-2,2932217	1,8565626	4,51579984	-0,3079737	
Dilution						
	X SOL	dil 1-15	UA			
Number of v	15	10	10			
R	1,34581324	1,21093988	1,24743371			
Std. Deviatio	0,07650696	0,03161713	0,05759084			
Relative Erro	0,05684813	0,02610958	0,04616745			
S/N	39,7599505	-14,748932				
Absolute Erro	4,09589433	-1,0660091				
